# Mechanisms and modulators of cognitive training gain transfer in cognitively healthy aging: study protocol of the AgeGain study

**DOI:** 10.1186/s13063-018-2688-2

**Published:** 2018-06-27

**Authors:** Dominik Wolf, Oliver Tüscher, Stefan Teipel, Andreas Mierau, Heiko Strüder, Alexander Drzezga, Bernhard Baier, Harald Binder, Andreas Fellgiebel, the German AgeGain study group, Andreas Fellgiebel, Oliver Tüscher, Bernhard Baier, Dominik Wolf, Bianca Kollmann, Florian Fischer, Alexandra Sebastian, Heiko Strüder, Andreas Mierau, Kristel Knaepen, David Riedel, Alexander Drzezga, Stefan Teipel, Katharina Brüggen, Judith Henf, Esther Lau, Harald Binder

**Affiliations:** 1 Department of Psychiatry and Psychotherapy, University Medical Center Mainz, Untere Zahlbacher Straße 8, 55131 Mainz, Germany; 2 Department of Psychiatry and Psychotherapy and German Resilience Center (DRZ), University Medical Center Mainz, Untere Zahlbacher Straße 8, 55131 Mainz, Germany; 3 Clinic of Psychosomatic and Psychotherapeutic Medicine, German Center for Neurodegenerative Diseases (DZNE), University Medical Center Rostock, Gehlsheimer Straße 20, 18147 Rostock, Germany; 4 German Sport University Cologne, Institute of Movement and Neurosciences, Am Sportpark Müngersdorf 6, 50933 Cologne, Germany; 5 Institute of Movement and Neurosciences, German Sport University Cologne, Am Sportpark Müngersdorf 6, 50933 Cologne, Germany; 6 Department Of Nuclear Medicine, University Clinic Cologne, Kerpener Straße 62, 50937 Cologne, Germany; 7 Edith-Stein-Fachklinik, Wiesenstraße 25, 76887 Bad Bergzabern, Germany; 8 Department of Neurology, University Medical Center Mainz, Langenbeckstr.1, 55131 Mainz, Germany; 9 Faculty of Medicine and Medical Center – University of Freiburg, Institute for Medical Biometry and Statistics, Stefan-Meier-Straße 26, 79104 Freiburg, Germany

**Keywords:** Cognitive training, Transfer of training gains, Normal aging, Neurobiological mechanisms and modulators of transfer, Physical training

## Abstract

**Background:**

Cognitively healthy older people can increase their performance in cognitive tasks through training. However, training effects are mostly limited to the trained task; thus, training effects only poorly transfer to untrained tasks or other contexts, which contributes to reduced adaptation abilities in aging. Stabilizing transfer capabilities in aging would increase the chance of persistent high performance in activities of daily living including longer independency, and prolonged active participation in social life. The trial AgeGain aims at elaborating the physiological brain mechanisms of transfer in aging and supposed major modulators of transfer capability, especially physical activity, cerebral vascular lesions, and amyloid burden.

**Methods:**

This 4-year interventional, multicenter, phase 2a cognitive and physical training study will enroll 237 cognitively healthy older subjects in four recruiting centers. The primary endpoint of this trial is the prediction of transfer of cognitive training gains. Secondary endpoints are the structural connectivity of the corpus callosum, Default Mode Network activity, brain-derived neurotrophic factors, motor fitness, and maximal oxygen uptake.

**Discussion:**

Cognitive transfer allows making use of cognitive training gains in everyday life. Thus, maintenance of transfer capability with aging increases the chance of persistent self-guidance and prolonged active participation in social life, which may support a good quality of life. The AgeGain study aims at identifying older people who will most benefit from cognitive training. It will increase the understanding of the neurobiological mechanisms of transfer in aging and will help in determining the impact of physical activity and sport as well as pathologic factors (such as cerebrovascular disease and amyloid load) on transfer capability.

**Trial registration:**

German Clinical Trials Register (DRKS), ID: DRKS00013077. Registered on 19 November 2017.

**Electronic supplementary material:**

The online version of this article (10.1186/s13063-018-2688-2) contains supplementary material, which is available to authorized users.

## Background

### Scientific background

Cognitive health has been consistently quoted as significant for life quality by older people and is regarded as an important contributor to late-life functioning [1, 2]. Patterns of cognitive change show great variation in healthy aging [3]. Cognitive training might contribute to enhance or preserve cognitive skills in cognitively healthy older adults (HOA). While improvements in cognition through training have been reported frequently in HOA, the capability to transfer cognitive training gains decreases with age [4–8]. Ideally, cognitive training would not only improve the function of trained cognitive tasks but also of untrained tasks of the same or a different cognitive domain (i.e., transfer of training gains). There is little evidence for successful transfer of cognitive training effects in HOA [6, 9]. Moreover, although transfer has been the subject of research for many years [10] the neurobiological mechanisms underlying the complex capability of transfer are still unknown. We recently demonstrated successful transfer of logical reasoning training gains to fluid intelligence immediately after a 4-week training in 71% of the HOA (*N* = 29 out of 41), but this effect persisted only in a subgroup of 22% over a 3-month follow-up period (*N* = 9 out of 41) [11].

**Fig. 1:**
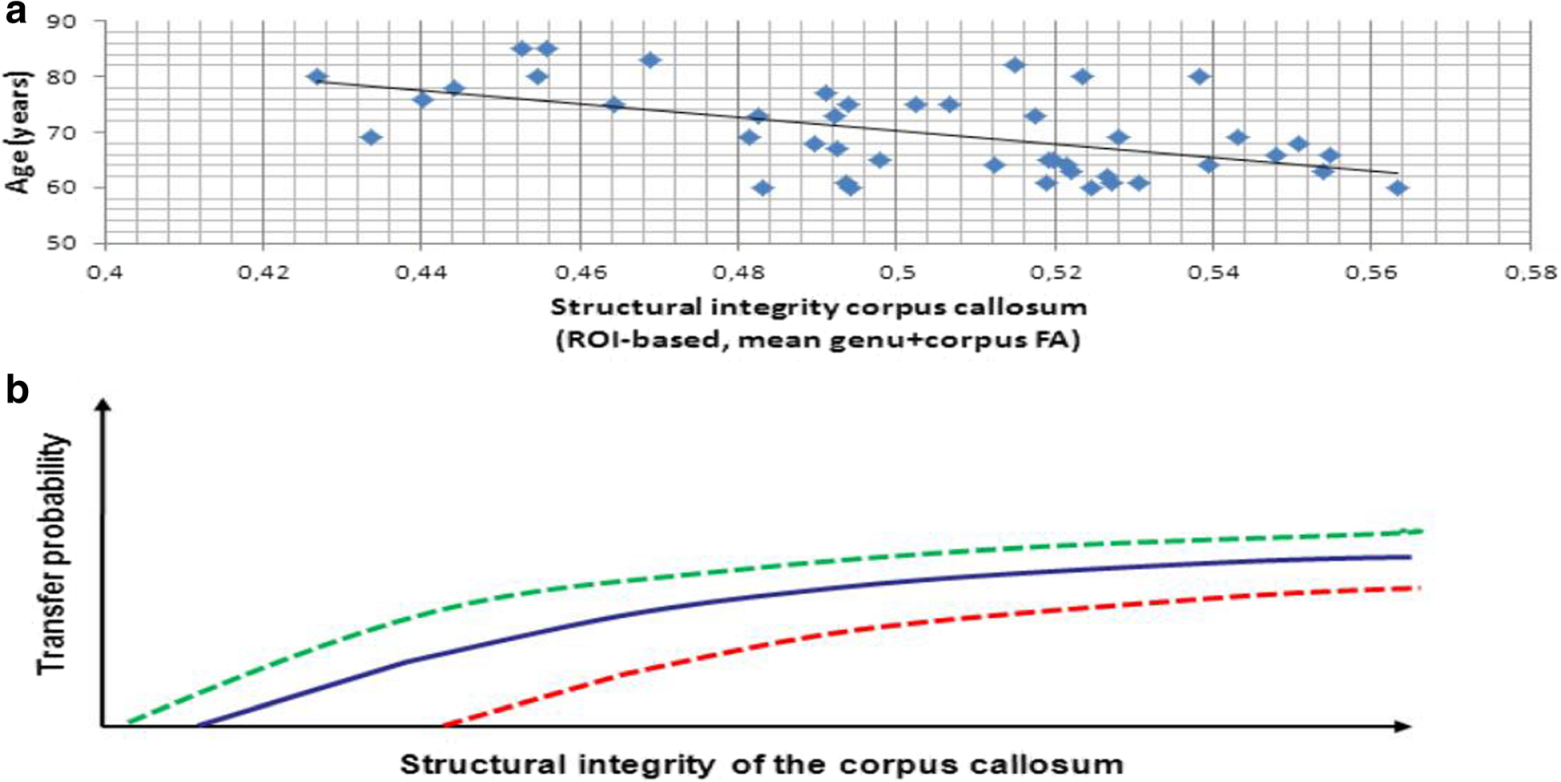
Age-relation of callosal structural integrity (**a**) and hypothetical association of callosal structural integrity with transfer probability in cognitively healthy elderly (**b**). **a** Fractional anisotropy (FA) of the genu and corpus of the corpus callosum decreases with age in cognitively healthy elderly [11]; higher FA values indicate better structural integrity. **b** Hypothetical probability of transfer decreases with decreased structural integrity of the corpus callosum

When we assessed structural underpinnings of successful transfer in this previous study [11], we focused on corpus callosum integrity as a structural prerequisite for successful bihemispheric cooperation, since cross-hemisphere processing results in better performance than within-hemisphere processing in complex cognitive tasks [12]. Since the callosal structural integrity is related to age (see Fig. 1a), and age-related disruption of corpus callosum microstructure in HOA impacts the efficiency of bihemispheric processing [13], we expected that associations between age and transfer might be moderated by the structural integrity of the corpus callosum. While short-term transfer was not related to structural integrity, stable transfer (ST) was predicted by the structural integrity/ connectivity of the corpus callosum [11]. Based on this preliminary evidence [11] we hypothesize that bihemispheric processing supported by intact corpus callosum integrity is a precondition of successful transfer in HOA (see Fig. 1a and b). Beside age, several other factors are likely to modulate corpus callosum integrity and other preconditions of successful bihemispheric processing and thereby facilitate or impair the transfer capability in HOA, such as physical activity or physical training, cerebral vascular or cerebral amyloid pathology, genetic factors of risk or resilience, and general intelligence [14, 15].

### Objectives

#### Primary objective

Four types of transfer training interventions have been described: strategy-based (e.g., logical reasoning), multimodal (complex, often social or lifestyle interventions), cardiovascular (i.e., physical training), and specific cognitive process-targeted training interventions (e.g., working memory) [16]. Transfer effects differ between transfer types but share a common feature: transfer effects decline with age [16]. To date, little is known about the neural mechanisms of transfer [16]. Specifically, the mechanisms of either maintenance or age-related decrease of transfer of cognitive training gains in HOA are largely unknown. Increased bihemispheric cooperation can reliably be seen with increasing processing demands in young adults and, as we showed, already on lower levels of cognitive demand in HOA [17]. This compensatory increase of bihemispheric processing in HOA is known as the concept of Hemispheric Asymmetry Reduction in Older Adults (HAROLD) [12]. This phenomenon could be the functional link between the observed association of transfer capabilities and structural integrity of the corpus callosum. Beyond pure compensation, HAROLD may serve as a mechanism enabling the structurally and functionally altered aged brain to use alternative neural circuitry and thereby to re-establish more efficient lateralized processing during learning [16]. Hence, increased bihemispheric cooperation/HAROLD, based on the structural integrity of the corpus callosum, may indeed be a functional mechanism mediating transfer capabilities in HOA.

**Fig. 2:**
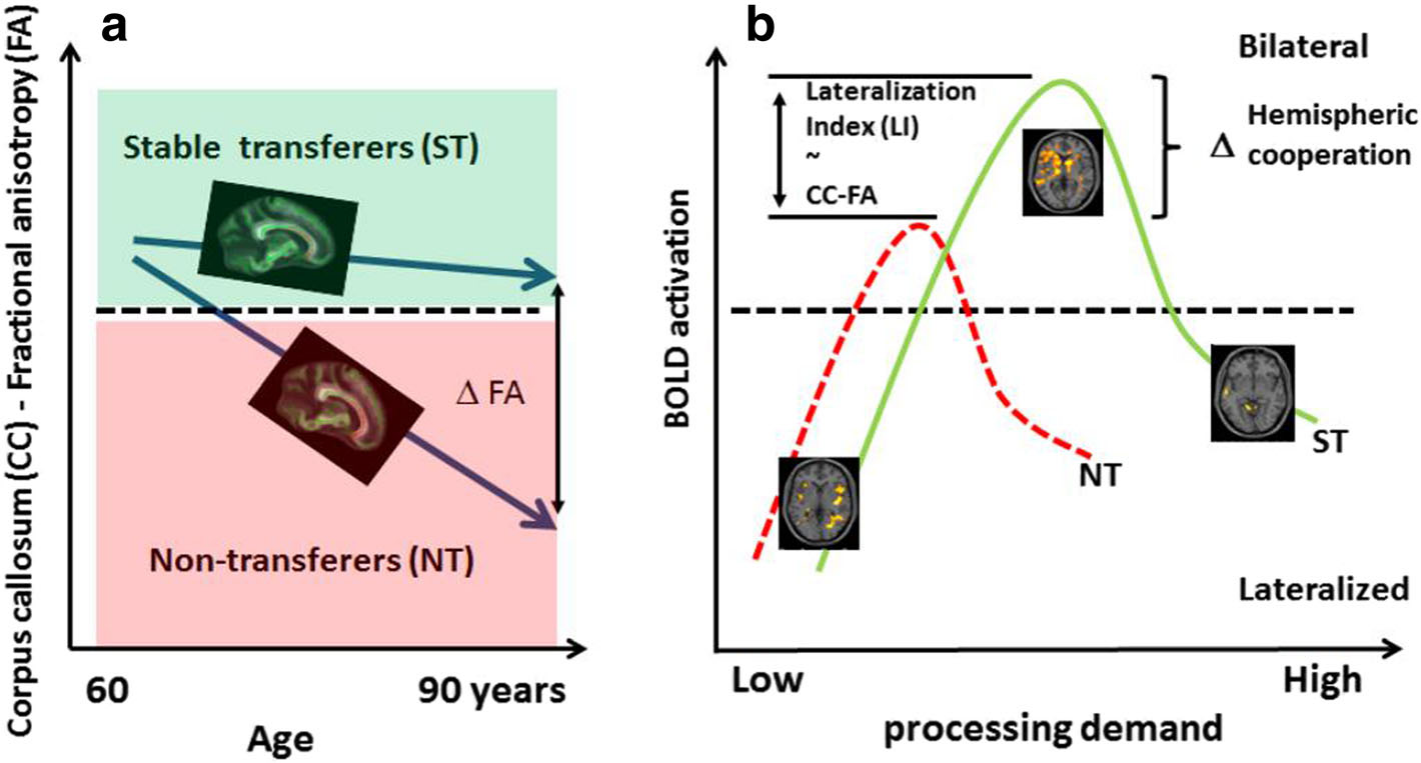
Joint models of brain structural (**b**) and functional (**a**) mechanisms for the explanation of transfer capability in healthy aging. **a** Fractional anisotropy (FA) of the genu and corpus of the corpus callosum decreases with age in cognitively healthy elderly; higher FA values indicate better structural integrity. Structural integrity of the corpus callosum predicts transfer capability determined by stable success (ST) versus non-transfer (NT) [11]. Categorial transfer was defined as an increase of fluid intelligence performance (transfer task) beyond the retest effect of untrained healthy elderly after successful training of logical reasoning skills [11]. Taken the corpus callosum structural integrity (FA) as surrogate of transfer capability, the model delineates a threshold of structural integrity (− − −) dividing ST and NT. Moreover, the model suggests that (e.g., z-standardized) FA values could be taken as dimensional predictors of the transfer amount in single subjects. **b** Increased hemispheric cooperation/HAROLD as measured by BOLD lateralization index [70] may mediate transfer capabilities in older adults since both are associated with the structural integrity of the corpus callosum. NT subjects may show less hemispheric cooperation compared to ST subjects at baseline thereby predicting less transfer success while both groups show the general pattern of lateralized to bilateral to disengagement of activity with increasing task demand [17]

Main research goal of the AgeGain study is to investigate the neurobiological mechanisms of transfer of cognitive training gains in detail by elaborating brain network capabilities representing transfer using a multimodal-neuroimaging approach in HOA. Specifically, the following primary hypothesis will be tested: Structural integrity of the corpus callosum (high / low) and bihemispheric cooperation (reduced / increased) will predict successful transfer of cognitive training gains in HOA. The association of corpus callosum integrity with transfer capability will be determined by diffusion-tensor imaging (DTI) measures of structural integrity / connectivity, mainly fractional anisotropy (FA) of the corpus callosum, see Fig. 2a. The association of bihemispheric processing and transfer capability will be determined using task-related functional magnetic resonance imaging (fMRI) (see Fig. 2b).

#### Secondary objectives

There is evidence that physical activity and cardiorespiratory fitness are associated with reduced brain tissue loss and reduced risk for cognitive impairment in aging humans [18, 19]. Moreover, aerobic exercise training increased gray and white matter volume in the prefrontal cortex [20] of older adults and led to significant improvements of executive function [21, 22]. Further, hippocampal and medial temporal lobe volumes were larger in older adults with greater cardiorespiratory fitness [19, 23], and larger hippocampal volumes mediated improvements in spatial memory [19]. Aerobic exercise training increases cerebral blood volume [24], perfusion [25] and the adult hippocampal volume. It has been suggested that the aforementioned structural changes are mediated by neurotrophins, in particular the brain derived neurotrophic factor (BDNF) [26]. We [27–30] and others [31, 32] demonstrated that exercise induces significant increases in levels of BDNF and other growth factors, and higher serum BDNF after aerobic training was associated with larger anterior hippocampal volume [33]. Using DTI, more recent studies measured brain structural integrity as a function of cardiorespiratory fitness and aerobic exercise training in HOA. Fractional anisotropy of the corpus callosum was reported to be positively correlated to cardiorespiratory fitness in HOA [34]. Thus, cardiorespiratory fitness might modulate age-related alterations of corpus callosum integrity. In addition, greater aerobic fitness derived from a walking program was associated with increases in white matter integrity of the frontal and temporal lobes, and improvement of memory performance [14]. Consistently, using resting state fMRI analyses, it has been shown that cardiorespiratory fitness is positively associated with functional connectivity in the Default Mode Network, and that this in part mediates improved performance on tasks requiring set-shifting, task switching, and spatial working memory [35]. Furthermore, 1 year of regular walking increased functional connectivity between parts of the frontal, posterior, and temporal cortices within the Default Mode Network and the Frontal Executive Network [35]. Of note, there is evidence that the beneficial effects of physical activity on cognitive performance is not limited to cardiorespiratory fitness but is also present after resistance [36–38] and coordination exercise training [39, 40]. Moreover, different types of physical training have been shown to affect different neurocognitive networks [40, 41]. We hypothesize that coordination exercise training positively modulates cognitive transfer abilities in HOA based on the following rationale: coordination training aims at improving the efficiency of complex body movements including eye-hand coordination, bimanual coordination, leg-arm coordination, and reactions to moving objects [40]. Such complex movements require bihemispheric interactions via the corpus callosum [42], and both, size and structural integrity of the corpus callosum typically change after sensorimotor skill acquisition [43]. These interactions are probably not limited to pure motor processes but rather include selective aspects of cognition necessary for adaptive motor behavior such as attention, inhibitory and facilitatory processes, working memory, and error-related processing. In line with this, it has been demonstrated that rather anterior corpus callosum projections to the prefrontal cortex than middle corpus callosum projections to the primary motor cortex predict bimanual motor learning [44].

In sum, aerobic exercise – and coordination exercise training are associated with positive structural and functional brain changes as well as improvements of cognitive performance in HOA. Moreover, cerebral regions targeted by these interventions are primarily involved in age-related cognitive decline including the corpus callosum, the hippocampus, frontal and temporal cortices, and the Default Mode Network. As a secondary outcome, we will determine if physical training, comprising aerobic and coordination components, modulates transfer of cognitive training gains in HOA. Moreover, we will determine the impact of baseline physical activity on transfer in HOA.

Subcortical cerebrovascular lesions reflected in white matter hyperintensities and cortical β-amyloid aggregation are highly prevalent and strongly age associated with central nevous system (CNS) pathologies [45]. Both findings are suspected to have a deleterious effect on neuronal function. White matter hyperintensities have been found to affect structural connectivity in the brain on the subcortical level [46]. Amyloid pathology, on the other hand, which is a hallmark of Alzheimer’s disease [47], has been discussed to affect regional synaptic function on the cortical level [48]. Although both findings have been associated with manifest cognitive decline or dementia, they are known to be present in a significant proportion of the cognitively healthy elderly, potentially leading to subclinical deficits [49, 50]. White matter hyperintensities frequently observed in the prefrontal cortex in clinically HOA have been linked to subclinical disturbances of executive function, whereas cortical amyloid deposition has been associated with a minor decline in memory function in this population [51]. Transfer capability represents a multidimensional cognitive function which requires executive and memory skills and presumably depends on highly efficient neuronal pathways including bihemispherical communication. Thus, the two mentioned pathologies are supposed to have a cumulative negative effect on transfer performance, even ahead of other cognitive symptoms. Consistently, as a further secondary outcome, the study aims at investigating white matter hyperintensities and cerebral amyloid burden as potential modulators of transfer of cognitive training gains in HOA.

We and others found that Default Mode Network activity is related to cognitive function and cognitive reserve in HOA [52, 53]. Default Mode Network activation during cognitive tasks was accompanied by less default suppression with greater task demand and less connectivity among Default Mode Network regions as well as increased frontal activation [54]. Thus, since cognitive reserve capacity as referring to Default Mode Network might also predict transfer cabilities, resting-state fMRI connectivity measurements will be also conducted [55]. Moreover, based on the comprehensive data survey the study aims at modeling and analyzing multimodal, high-dimensional datasets with respect to transfer prediction and to build a robust individual index of transfer likelihood.

All secondary outcomes are summarized in Table 1.

**Table 1:** Overview of secondary outcomes

Secondary objectives	To evaluate the predictive value of a preceding aerobic and coordination training for transfer of cognitive training gains in healthy older adults (HOA)
	To evaluate the predictive value of baseline physical activity on transfer in HOA. Baseline physical activity will be measured by a 1-week actigraphy and the Global Physical Activity Questionnaire (see “Measures” section below).
	To evaluate the predictive value of brain vascular lesion (as determined by T2-weighted magnetic resonance imaging (MRI), cortical amyloid burden (as determined by positron-emission tomography), higher default mode network activity (as determined by resting state functional MRI (fMRI) for transfer of cognitive training gains in HOA
	To model and analyze multimodal, high-dimensional datasets with respect to transfer prediction and to build a robust individual index of transfer likelihood

## Methods/design

### Study type

This is a longitudinal, interventional, parallel-group, multicenter, multimodal-imaging trial.

### Study design

In this 4-year parallel-group, multicenter, multimodal-imaging study, cognitively healthy elderly subjects will be enrolled by three recruiting centers in Germany: Mainz (University Medical Center Mainz - Department of Psychiatry and Psychotherapy), Rostock (University Medical Center Rostock, Clinic of Psychosomatic and Psychotherapeutic Medicine and German Center for Neurodegenerative Diseases (DZNE)), and Cologne (German Sport University Cologne and University Hospital Cologne – Department of Nuclear Medicine). The experimental protocol can be divided into five phases (see Table 2). In phase 1, inclusion and exclusion criteria will be assessed (screening). In phase 2 (pre-cognitive-training phase), participants will undergo baseline neuropsychological and physical activity assessment, MRI, positron-emission tomography (PET), and genetics. Thereafter, in phase 3 (cognitive-training phase), a cognitive training will be applied over a period of 4 weeks (with three 60-min-long training sessions per week). Directly following, in phase 4 (post-cognitive-training phase), participants will repeat the neuropsychological assessment to determine immediate transfer effects. Finally, in phase 5 (follow-up, after 3 month), a final neuropsychological assessment will be applied to determine ST effects. The study protocol follows the SPIRIT (Standard Protocol Items: Recommendations for Interventional Trials) recommendations (see Additional file 1).

**Table 2:**
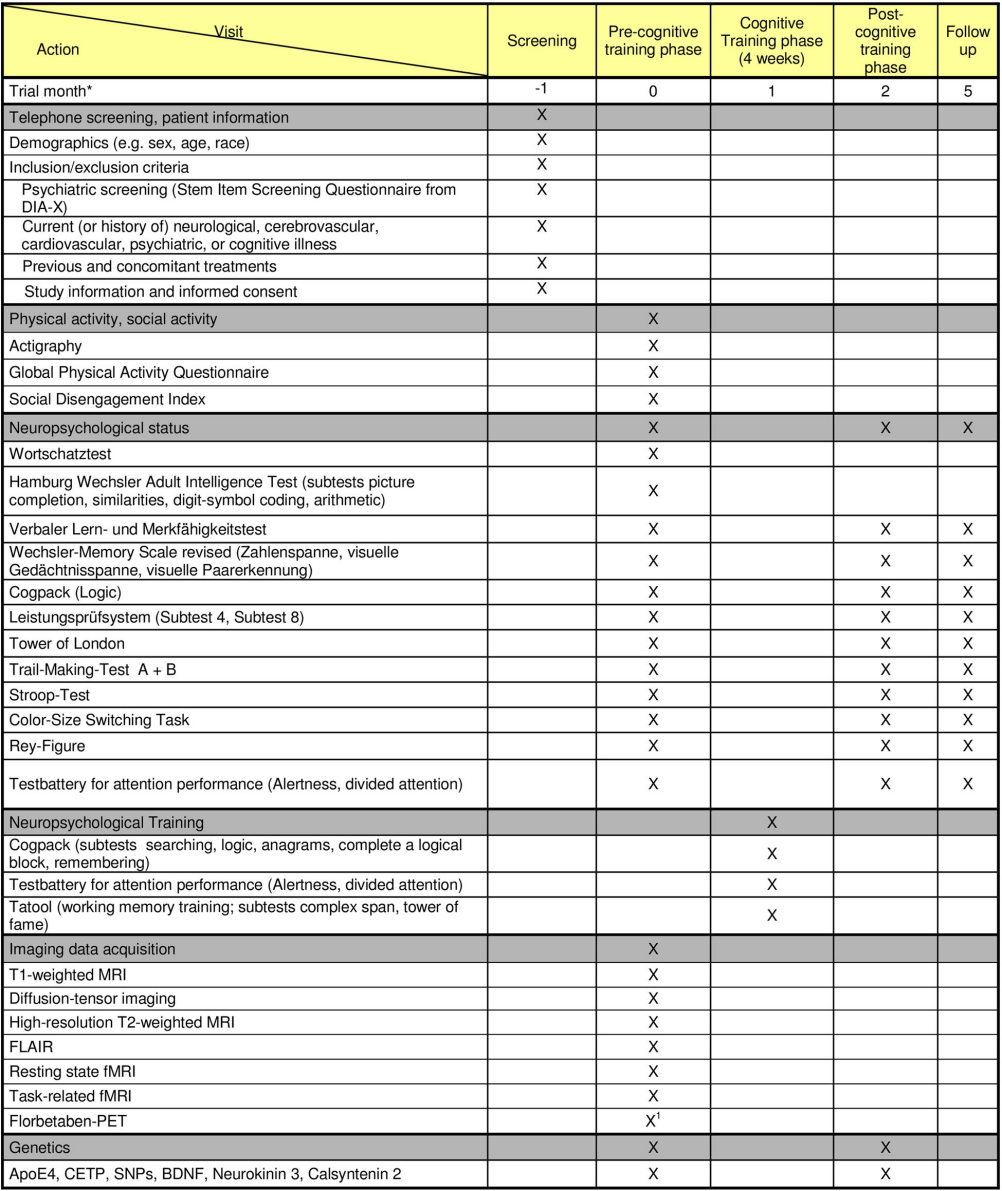
Trial schedule University Medical Center Mainz and University Rostock

^a^Schedule for each subject

^b^The Florbetaben-PET can be postponed to the post-cognitive-training phase or the follow-up phase

Abbreviations: *MRI*  magnetic resonance imaging, *fMR*I functional magnetic resonance imaging, *PET* positron-emission tomography, *ApoE4*  apolipoprotein E4, *CETP* Cholesteryl Ester Transfer Protein, *SNP*  single nucleotide polymorphism, BDNF brain-derived neurotrophic factor

To investigate the impact of a physical training (combined aerobic and coordination training, ACT) on transfer of cognitive training gains in HOA (secondary outcome), subjects recruited at the center in Cologne will undergo an extended experimental protocol with two additional phases prior to the pre-cognitive-training phase (pre-ACT-training phase, ACT-training phase). The primary protocol (as shown in Table 3) will be complemented by a 20-week physical training period and an evaluation of the physical fitness, as well as an additional MRI scan evaluation (see Table 3).

**Table 3:**
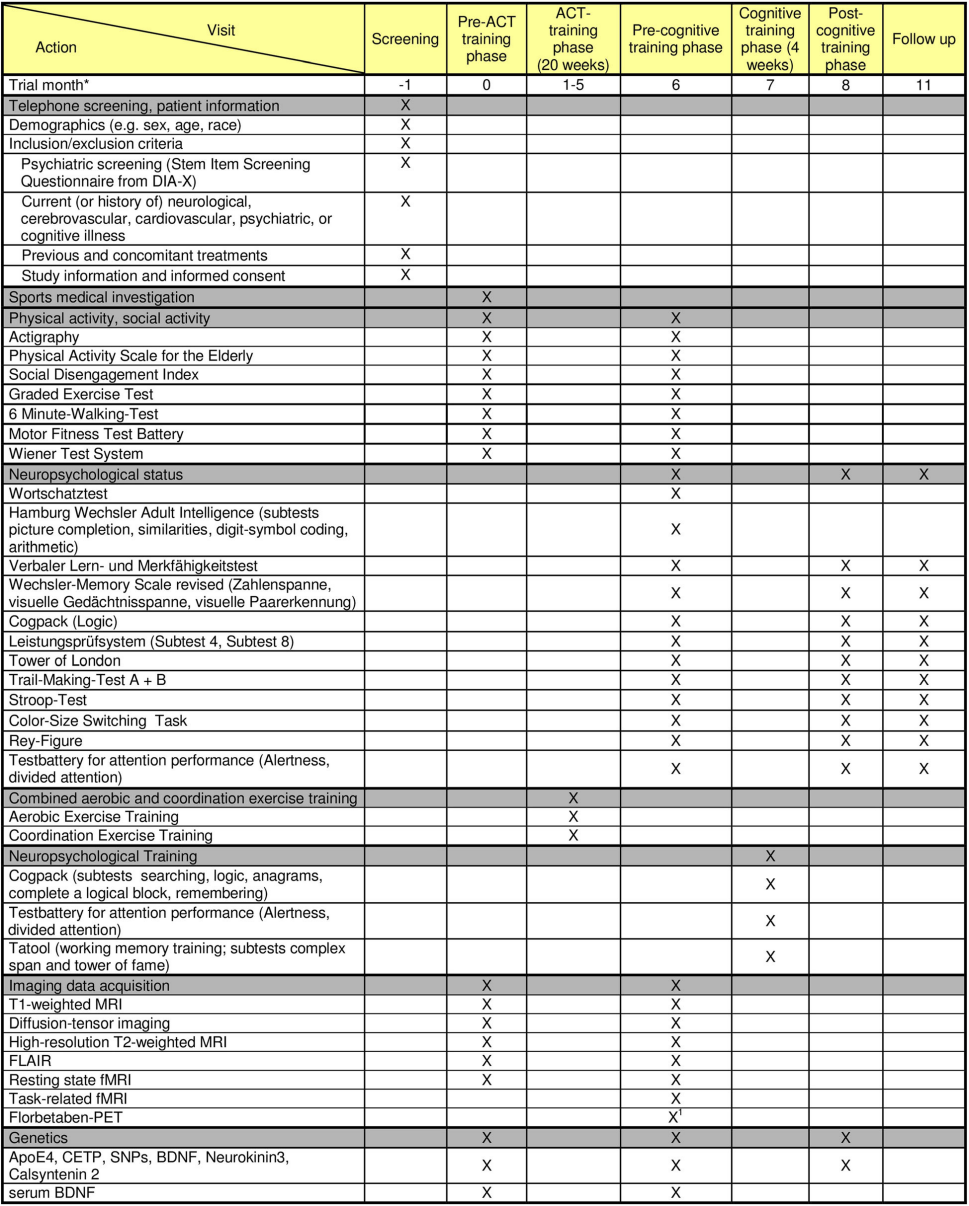
Trial schedule German Sport University Cologne/University Hospital Cologne

^a^Schedule for each subject

^b^The Florbetaben-PET can be postponed to the post-cognitive-training phase or the follow-up phase

Abbreviations: *MRI*  magnetic resonance imaging, *PET* positron-emission tomography, *ApoE4* apolipoprotein E4; *CETP*  Cholesteryl Ester Transfer Protein, *SNP* single nucleotide polymorphism, *BDNF*  brain-derived neurotrophic factor

### Population

#### Sample size

It is planned to include 237 cognitively healthy elderly subjects (aged 60 years and older) in total distributed over the three trial sites, satisfying the statistical power requirements for different research questions, as described below. Paticipants will be recruited by local newspaper announcements and flyers.

For the investigation of the primary objective, a sample size of 160 subjects has been determined based on a power calculation (see “Statistics”). Assuming a dropout rate of about 15%, the recruitment of subjects will be distributed as follows: Mainz: *N* = 60, Rostock: *N* = 60, German Sport University Cologne/University Hospital Cologne: *N* = 72.

For the investigation of the predictive value of a preceding ACT for the transfer of cognitive training gains in HOA (secondary objective), all 72 subjects recruited at the German Sport University Cologne/University Hospital Cologne (who are also part of the investigation of the primary outcome) will undergo an extended experimental protocol, including ACT followed by the cognitive training. The sample size of 72 subjects has been determined based on a power calculation (see “Statistics”). Since, so far, no evidence has been published showing that physical activity may have an impact on transfer via mechanisms other than brain structure and function, it is assumed that the preceding physical training in subjects recruited in Cologne will not confound the investigation of the primary objective.

To minimize/avoid bias, the following control subjects will be included: 15 HOA will be enrolled in Cologne undergoing the standard experimental protocol without ACT to assure that the net effect of the cognitive training does not differ from the effects at the other trial sites. To control performance alterations in the neuropsychological examinations for retest effects, an additional sample of 30 HOA, generated in Mainz (*N* = 10), Rostock (*N* = 10), and German Sport University Cologne/University Hospital Cologne (*N* = 10), will undergo the repeated neuropsychological examinations without cognitive training.

Within each trial site, participants will be distributed randomly to experimental or control groups, stratified centrally by the trial coordinator at the trial site University Medical Center Mainz. The study code is centrally managed by this trial site. Participants will be enrolled by their respective trial site.

#### Inclusion criteria (for all trial sites)

The following inclusion criteria will be applied:

- Age ≥ 60 years
- Ability of subject to understand character and individual consequences of clinical trial
- Signed and dated informed consent must be available before start of any procedures
- Sufficient mobility and motivation to be able to participate in the examinations

#### Exclusion criteria (for all trial sites)

- Incapability of giving consent
- Current (or history of) psychiatric illness
- Current (or history of) neurological or cerebrovascular illness, brain lesions
- Current (or history of) cardiovascular disease (i.e., myocardial infarct, peripheral arterial disease)
- Secondary disorders restricting individuals’ physical capacity (i.e., chronic obstructive pulmonary disease, rheumatism, osteoarthritis, bone fractures)
- Current (or history of) cognitive illness
- Diabetes types 1 and 2
- Intake of medications that may influence cognitive performance
- Insufficient German language skills
- Participation in other clinical trials during the present clinical trial or within the last month
- MRI contraindication (pacemaker, metal implants, tattoos, permanent-make-up, chochlear implant, medication pump, acupuncture needles)

### Measures

#### Assessment of inclusion/exclusion criteria

- Telephone screening
  Demographics, psychiatric screening (diagnostic expert system for psychiatric disorders – Stamm Screening Interview [56], International Diagnostic Checklists for ICD − 10 and DSM-IV [57]
- Study information and informed consent
  General study information and informed consent, MRI information and informed consent, PET information and informed consent, genetics information and informed consent

#### Neuropsychological examination at baseline (NP I)

- Intelligence
  Wortschatztest [58], Hamburg-Wechsler Intelligence Test for adults-revised – subtests: picture completion, similarities, block design, arithmetic [59]
- Memory
  Verbaler Lern- und Merkfähigkeitstest [60], Wechsler-Memory Scale-revised – subtests: digit span, block span (forward and backward, respectively) [61], Cogpack – subtest: remembering [62]
- Executive function
  Leistungsprüfsystem (comparable to the Raven Matrices): subtest 4 [63], Tower of London [64], Trail-Making Test B [65], Cogpack – subtest: reasoning [62].
- Stimulus interference
  Computerized version of the Stroop Test
- Information processing speed
  Trail-Making Test A [65], Hamburg-Wechsler Intelligence Test for adults-revised: digit-symbol substitution test [59]
- Visual construction
  Ray-Osterrieth Complex Figure Test – copy [66].
- Attention
  Test battery for attention performance – subtests: alertness, divided attention

#### Neuropsychological examination immediately after cognitive training (NP II)

In the second neuropsychological examination (NP II, post-training phase), the same test battery as in the baseline examination (NPI) will be applied. However, the tests measuring intelligence will only be applied once (at NP I). If available, we will apply parallel test versions to avoid retest effects.

#### Neuropsychological examination after 3 months (NP III)

In the third neuropsychological examination (NP III, follow-up), the same test battery as in NP II will be applied. If available, we will apply parallel test versions to avoid retest effects.

#### Neuropsychological training

- Executive functions, memory, information processing speed
  Cogpack: Cogpack is a computerized cognitive training and testing program. The subtests comparisons, searching, logic, anagrams, complete a logical block, and remembering will be applied
- Alertness
  Attention capacities will be trained using the test battery for attention performance, which permits us to assess/train a variety of attentional aspects. The subtests alertness and divided attention will be trained.
- Working memory
  The computerized training software TATOOL [67] will be used to apply a working memory training designed in accordance to Batian et al. [68]

#### Physical activity examination

- Actigraphy
  Objective physical activity, sleep/wake and energy expenditure measurement solution. The portable wristband (GeneActive, Kimbolton, UK) uses a three-axis accelerometer, a heat flux sensor, a galvanic skin-response sensor, a skin-temperature sensor, and a near-body ambient temperature sensor to capture data for 1 week
- Global Physical Activity Questionaire
  The Global Physical Activity Questionaire covers several components of physical activity typical of an elderly population. The score takes into account self-reported occupational, household and leisure activities items over a 1-week period (World Health Organization (WHO), http://www.who.int/chp/steps/GPAQ/en/)

#### Fitness examinations

- Cardiovascular fitness
  Cardiovascular fitness will be assessed using a modified, graded, exercise-testing protocol in the field. Endurance capacity will be estimated on the basis of walking/running speed, the corresponding concentration of blood lactate and the perceived exertion of each participant.
  Additionally, the maximum oxygen uptake (VO_2_max) and the peak oxygen uptake (VO_2_peak) will be estimated using the 6-minute Walking Test.
- Motor fitness
  Gross motor fitness will be assessed using a test battery comprising the following basic motor skills:
  - Static and dynamic balance
  - Short Physical Performance Battery
  - Feet tapping
  - Hand tapping
  - Kasten-Bumerang Test
  - Movement-Coordination Test
  - Agility Test
- Fine motor fitness will be assessed using the following subtests of the Vienna Test System:
  - Motor performance series
  - Sensomotor coodination
  - Spatial orientation
  - Reaction time
  - Response inhibition
  - Time/movement anticipation

#### Imaging data acquisition

**Fig. 3:**
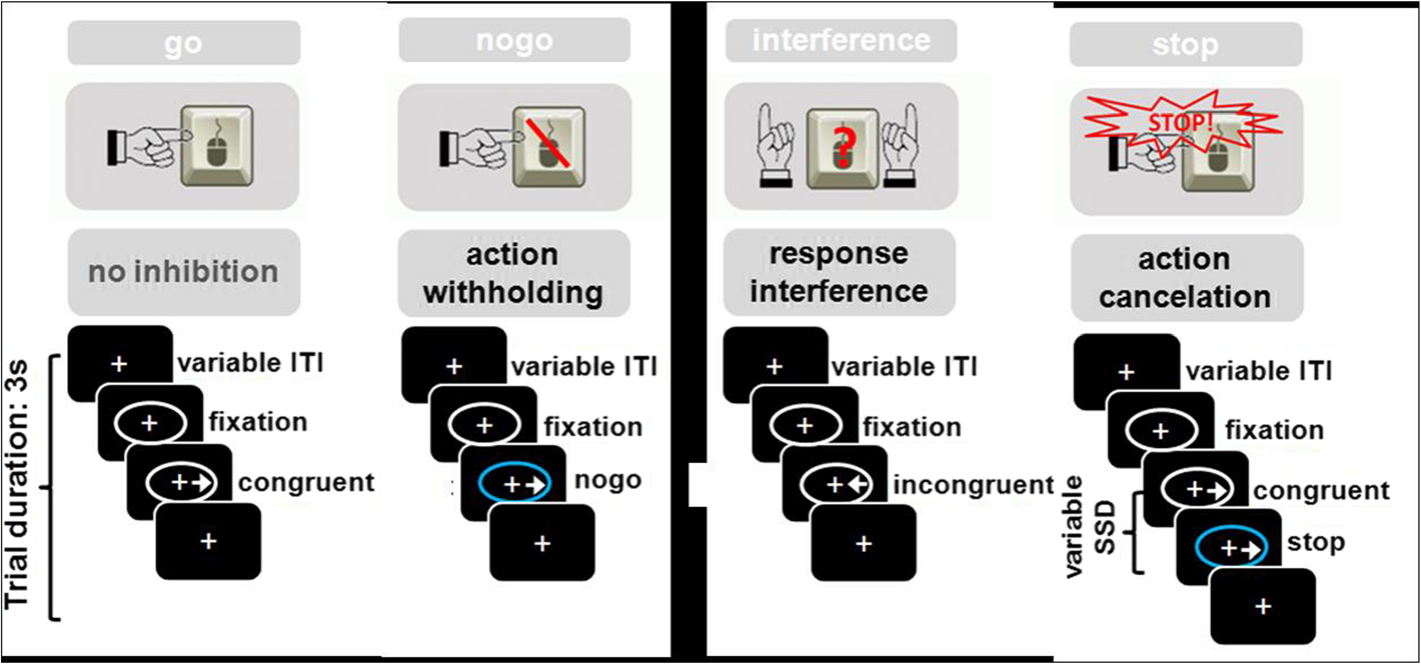
The Hybrid Response Inhibition task. Participants are asked to press a button corresponding to the pointing direction of an arrow. Go trials consist of congruent trials; inhibition trials consist of incongruent trials (interference inhibition), occurrence of a no-go stimulus (blue ellipse; action withholding), or of a stop-signal (blue ellipse after a varying stop-signal delay; action cancelation)

- Structural MRI
  DTI, T1-weighted structural MRI, FLAIR, high-resolution T2-weighted structural MRI
- Functional MRI
  Resting-state MRI, task-related fMRI. For task-related fMRI, participants will perform three runs of the Hybrid Response Inhibition task [69]. Using identical visual stimulus material the Hybrid Response Inhibition task assesses three subcomponents of response inhibition: response interference, action withholding, and action cancelation (Fig. 3). Stimuli will be presented in the center of the screen. Participants will be asked to perform a button press according to the pointing direction of an arrow and to refrain from a button press whenever the ellipse surrounding the arrows turns blue (nogo−/stop trials). Event-related fMRI data acquisition will be performed using standard echo planar imaging sequences with whole-brain coverage and isotropic voxels. SPM 12 (http://www.fil.ion.ucl.ac.uk/spm) will be used to conduct all image preprocessing and statistical analyses. In addition to standard general linear model analyses, a BOLD lateralization index [70] will be computed for each subcomponent separately. The lateralization index is hypothesized to mediate transfer capabilities in older adults and to be associated with the structural integrity of the corpus callosum.
- PET
  The amyloid tracer [18F]Florbetaben will be applied via a venous cannula. Subjects will be instructed to void their bladder to allow rapid excretion of unbound radioactivity. Subjects will be placed on the scanner approximately 70 min after injection. At 80 min p.i. (post injection) two low-dose computer tomography scans will be acquired for position and attenuation correction. At 90 mins p.i., the PET acquisition will be initiated. PET data will be acquired for 20 min. After the scan the subject will be asked about their well-being and instructed to void their bladder to accelerate excretion of radioactivity. Subjects will be instructed to minimize contact with small children and pregnant women for 12 h after tracer injection. The entire examination, including preparation and scanning procedures, can be finished within 3–3.5 h.

#### Genetics

Two samples of 5 ml ethylenediamine tetraacetic acid blood will be collected from participants at the pre-training phase and at the post-training phase in Mainz and Rostock and at the pre-ACT-training phase, post-ACT-training phase, and post-cognitive-training phase in Cologne. Blood samples will be kept on ice for a maximum of 15 min and subsequently stored at − 80 °C in the recruiting centers. Frozen blood samples will be shipped on dry ice to the Laboratory of Molecular Genetics, Institute of Human Genetics, University Medical Center Mainz. There, deoxyribonucleic acid (DNA) extraction from one of the frozen blood samples taken at the pre-training phase will be performed on a chemagic Magnetic Separation Module I using the chemagic DNA Blood Kit special for the automated isolation of DNA from 5 ml whole blood followed by quantification of DNA on a NanoDrop 2000UV-Vis spectrophotometer. Genotyping for the single nucleotide polymorphisms rs7412 and rs429358 in the apolipoprotein E gene, rs5882 and rs1800775 in the *Cholesteryl Ester Transfer Protein* gene, rs6265 in the *BDNF* gene, rs2765 in the *Neurokinin.3 Receptor* gene, rs6439886 in the *Calsyntenin 2* gene, and rs17070145 in the *Kidney and Brain Expressed Protein* gene will be carried out from extracted genomic DNA samples using polymerase chain reaction followed by pyrosequencing on a Pyromark Q96 ID Instrument using PyroMark Gold Q96 SQA Reagents and the Pyromark Q96 ID software. The second blood sample taken at the pre-training phase and both blood samples taken at the post-training phase will be stored at − 80 °C for prospective analyses of training-induced epigenetic and transcriptome changes. Blood samples will be stored in secured rooms at the Institute of Human Genetics of the University Medical Center Mainz for a maximum of 60 years.

### Statistics

#### Sample size

Primary outcome: the analyses will focus on training gain transfer (defined as a binary criterion for each subject) and simultaneously incorporate measures of corpus callosum integrity and bihemispheric processing. The sample size calculation was performed based on main effects analyses where the effects of both measures will be considered as main effects. Assuming a transfer rate of cognitive training gains of about 15% for participants with low corpus callosum integrity, and a transfer odds ratio of 3 for participants with high corpus callosum integrity, a sample size of 160 provides a power of 0.8 at a significance level of 0.05 for a Wald Test in a logistic regression model. Assuming a dropout rate of about 15%, a total of 192 participants need to be included.

Secondary outcome: for evaluating physical training as a potential modulator of cognitive training gain transfer, ACT-induced fitness changes will be analyzed with respect to changes of corpus callosum integrity, as well as with respect to effects on transfer. The former analysis corresponds to considering the correlation between fitness changes and integrity changes. For the latter, an interaction between corpus callosum integrity and fitness changes with respect to transfer will be considered in a logistic regression model. Such an interaction can be expressed as a correlation between fitness changes and the degree of transfer in a group with low/high corpus callosum integrity. Based on the results by Voss et al. [14], we assume a correlation of about 0.43 between both measures. At a significance level of 0.05, 30 individuals per (equally sized) integrity group are needed to detect such a correlation with a power of 0.8. As a result, ACT needs to be performed for 60 individuals in total. Assuming a dropout rate of about 15%, 72 subjects at the German Sport University Cologne will undergo the extended experimental protocol.

#### Definition and analysis of primary endpoint

The primary endpoint is defined as the dichotomous outcome training gain transfer (Yes/No). Successful transfer will be defined as: (1) Performance improvement in a logical reasoning training task from the second to the last training session, (2) performance improvement in an untrained fluid intelligence transfer task (Leistungsprüfsystem, subtest 4) from the pre-cognitive-training phase to the post-cognitive-training phase, and (3) maintenance of the performance in the transfer task from post-cognitive-training phase to follow-up. Transfer task performance alterations have to be numerically greater than the mere retest effect of an untrained control group.

The primary endpoint will be analyzed using a logistic regression with measures of corpus callosum integrity and bihemispheric processing as independent variables. As a primary analysis, the effects of both measures will be considered as main effects. In subsequent analyses, we will take into account that bihemispheric processing may not be necessary for all participants, whereby strong training performance might specifically indicate such participants. This will be incorporated into the analyses by interaction terms and by allowing for non-linear effects. The models will be adjusted for age. In the primary analysis subjects with missing values will be excluded. Sensitivity analysis will be performed to evaluate the impact of the missing values.

The primary population is the intention-to-treat population. As a sensitivity analysis, an as-treated analysis will be performed. To investigate the center effects, center number will be additionally incorporated into the models.

#### Analysis of secondary endpoints

For evaluating physical training as potential modulator of cognitive training gain transfer, correlations between fitness changes and integrity changes of the corpus callosum will be applied. Moreover, an interaction between corpus callosum integrity and fitness changes with respect to transfer will be considered in a logistic regression model. Such an interaction can be expressed as a correlation between fitness changes and the degree of transfer in a group with low/high corpus callosum integrity.

To evaluate the predictive value of brain vascular lesion, cortical amyloid load, higher Default Mode Network activity, and genetics for transfer of cognitive training gains in HOA, a logistic regression model with transfer as the dependent variable is used. All effects of the potential predictors are included as main effects. Additionally, to adjust for ACT, ACT and all interactions with ACT are included in the model as independent covariates.

To determine the impact of ACT on the structural integrity of the corpus callosum, Default Mode Network activity, BDNF, motor fitness, and VO_2_peak/VO_2_max, logistic regression models for these variables are fitted. ACT will be included as an independent variable. Additionally, the model will be adjusted for all other variables and the interactions with ACT. For example, the model for structural integrity of the corpus callosum will include ACT, Default Mode Network activity, BDNF, motor fitness, VO_2_peak/VO_2_max and all interactions with ACT as independent covariates.

Similar models will be fitted to examine the association between baseline physical activity and the structural integrity of the corpus callosum, Default Mode Network activity, and BDNF. Here, baseline physical activity and all possible interactions with baseline physical activity will be additionally included as independent covariates.

To build a robust individual index of transfer likelihood based on all potential predictors and interactions, a multimodal, high-dimensional dataset needs to be analyzed. For this purpose a regularized logistic regression for transfer will be performed including all potential predictors and relevant interactions. Specifically, to automatically select relevant covariates, a componentwise likelihood boosting approach will be used. To better detect potentially complex patterns in the multimodal data that are linked to transfer, a deep learning approach will be employed; specifically, deep Boltzmann machines [71]. This specific approach will potentially allow for the detection of non-linear relations and interactions while still providing type 1 error control, and results in an integrated statistical model for transfer prediction.

### Data management

#### Responsibilities

The data management in each trial center during the trial will be conducted by one study member of the respective trial site (specified before the start of data collection). The trial coordinator is authorized to contact trial centers to monitor the data management.

#### Data collection

An electronic Case Report Form (eCRF) will be provided for each subject. All trial data will be documented in the subject’s source data and in the eCRF. The principal investigators of the trial sites or designated representatives are responsible for ensuring that all sections of the eCRFs of their trial sites are correctly completed and that entries can be verified against source data. All changes in an eCRF entry will be tracked. The investigator, or a designated representative, should complete the eCRF pages as soon as possible after the information is collected. Any outstanding entries must be completed immediately after the final examination. An explanation should be given for any missing data.

#### Data handling

After completion of data entry all data will be collected at the University Medical Center Mainz. The access for data entry will be blocked and checks for plausibility, consistency, and completeness of the data will be performed. Based on these checks, queries will be produced. Any missing data or inconsistencies will be reported back to the respective site and clarified by the responsible investigator. If no further corrections are to be made in the database it will be declared closed and used for statistical analysis. The data checks will be done by the trial coordinator or a designated representative at the University Medical Center Mainz.

### Assessment of safety

#### Assessment of adverse events (AEs) by investigator

Subjects must be carefully monitored for AEs by the investigator. The intensity of the AEs and the causal relation to trial medication and/or procedures are to be assessed.

The intensity of an AE will be assessed by the investigator as follows:

- Mild: temporary event which is tolerated well by the subject and does not interfere with normal daily activities
- Moderate: event which results in discomfort for the subject and impairs their normal activity
- Severe: event which results in substantial impairment of normal activities of the subject

The assessment of the relationship of an AE to trial procedures is a clinical decision based on all available information at the time of the completion of the eCRF. The investigator will evaluate the causal relationship of each adverse event with the trial procedures according to modified criteria of the WHO 1991.

#### Documentation of AEs and follow-up

All AEs (whether serious (SAE) or non-serious) reported by the subject or detected by the investigator will be documented on the “Adverse Event Page” of the eCRF. If an AE is serious, the investigator must complete, in addition to the “Adverse Event Page”, a “Serious Adverse Event Form” at the time the SAE is detected. SAEs are required to be reported by the investigator to the sponsor immediately (i.e., no more than 24 h after learning of the event).

All subjects who experience AEs, whether considered associated with the use of the investigational products or not, must be monitored to determine the outcome. The clinical course of the AE will be followed up according to accepted standards of medical practice, even after the end of the period of observation, until a satisfactory explanation is found or the investigator considers it medically justifiable to terminate follow-up, but no longer than 90 days after the end of the trial.

### Ethical and legal aspects

#### Good Clinical Practice

The procedures set out in this trial protocol, pertaining to the conduct, evaluation, and documentation of this trial, are designed to ensure that all persons involved in the trial abide by the quality standards of Good Clinical Practice and the ethical principles described in the Declaration of Helsinki. The trial will be carried out in accordance with all applicable local legal and regulatory requirements.

#### Patient information and informed consent

Before being enrolled into the clinical trial, the subject must consent to participate after being fully informed about the nature, scope, and possible consequences of the clinical trial. The documents must be in a language understandable to the subject and must specify who informed the subject. A copy of the signed informed consent document must be given to the subject. The investigator will retain the original signed consent document. The investigator will not undertake any measures specifically required only for the clinical trial until valid consent has been obtained. After reading the informed consent document, the subject must give consent in writing. The subject’s consent must be confirmed by the personally dated signature of the subject and by the personally dated signature of the person conducting the informed consent discussions.

#### Confidentiality

The name of the subject and other confidential information are subject to medical professional secrecy and the regulations of the German Data Protection Act (Bundesdatenschutzgesetz). The name of the subjects and other confidential information will not be supplied to the sponsor. During the clinical trial, subjects will be identified solely by means of an individual identification code (e.g., subject number, randomization number). Trial findings stored on a computer will be stored in accordance with local data protection law and will be handled in strictest confidence. For protection of these data, organizational procedures are implemented to prevent distribution of data to unauthorized persons. The appropriate regulations of data legislation will be fulfilled in their entirety. The investigator will maintain a personal subject identification list (subject numbers with the corresponding subject names) to enable records to be identified.

#### Approval of trial protocol

The study protocol was approved by the local Ethics Committees of all three trial sites (Mainz: Ethics Commission of the Landesärztekammer Rheinland-Pfalz; Cologne: Ethics Commission of Cologne University’s Faculty of Medicine; Rostock: Ethics Commission of the Rostock University’s Faculty of Medicine). The reference number of the ethics approval of the main trial site Mainz is 837.385.15 (10153). The study was registered at the German Clinical Trials Resgister (ID: DRKS00013077).

### Publication policy

Any publication of the results, either in part or in total (articles in journals or newspapers, oral presentation, etc.) by the investigators, their representatives, or by the sponsor, will require the approval of all principal investigators of the trial (Prof. Dr. Andreas Fellgiebel, Prof. Dr. Oliver Tüscher, Dr. Andreas Mierau, Prof. Dr. Alexander Drzezga, Prof. Dr. Stefan Teipel, Prof. Dr. Harald Binder). It is planned to publish the results of the trial as original articles in appropriate journals as well as to present the results at congresses.

## Discussion

Knowledge on the mechanisms and possibly modifiable modulators of transfer of cognitive training gains is urgently needed to design future intervention programs for the promotion of cognitive health including lifelong learning in aging. Today, neuroimaging techniques allow a reliable and valid investigation of neuronal mechanisms of transfer based on structural and functional brain network surrogates directly in humans. For this important purpose, the AgeGain Consortium combines all relevant multidisciplinary expertise, including cognitive training and transfer assessments, conduction of multimodal neuroimaging, physical training, and the assessment of subclinical brain pathology that occurs frequently and is known to significantly contribute to incident decline of cognitive function in HOA. As shown by recent publications [11, 17, 55, 72, 73], the individual research groups assembled in AgeGain are already focusing on these research questions and the first successful European collaborations have been established. The formation of the consortium will enable the development of a strong partnership of basic clinical research in HOA.

The AgeGain study should provide important information for the determination of transfer likelihood in older people, and thus for the identification of HOA, who will most benefit from cognitive training. Findings of this trial should contribute to a better understanding of the neurobiological mechanisms of transfer in aging and will help determining the impact of physical activity and sport as well as of pathological factors (such as cerebrovascular disease and amyloid load) on transfer capability. Specifically, the trial will contribute to an increased understanding of the association of corpus callosum integrity as a structural measure and bihemispheric cooperation as a functional measure (as well as their interaction) with transfer capabilities in older adults. Beyond that, the trial should add important information about the importance of HAROLD for learning in old age. The study results should have a strong impact on future clinical, basic, and healthcare research evaluating lifestyle and training strategies to maintain successful and lifelong learning. A limitation of the study is that it is restricted to the investigation of neurobiological mechanisms of transfer on a macrostructural level. Thus, it will not provide information about microstructural neurobiological mechanisms of transfer.

## Trial status

The study is currently in the recruitment phase. Recruitment began in July 2016.

Protocol version: 1.3 (06–21-2016).

## Additional file

Additional file 1: Standard Protocol Items: Recommendations for Interventional Trials (SPIRIT) 2013 Checklist: recommended items to address in a clinical trial protocol and related documents*. (DOC 121 kb)

## Abbreviations

ACT: Combined aerobic and coordination training
AE: Adverse event
APOE: Apolipoprotein E
BDNF: Brain-derived neurotrophic factor
CETP: Cholesteryl ester transfer protein
DTI: Diffusion-tensor imaging
eCRF: Electronic Case Report Form
FA: Fractional anisotropy
fMRI: Functional magnetic resonance imaging
GPAQ: Global Physical Activity Questionnaire
HAROLD: Hemispheric Asymmetry Reduction in Older Adults
HOA: Healthy older adult
MRI: Magnetic resonance imaging
NP: Neuropsychological examination
NT: Non-transfer
PET: Positron-emission tomography
SNP: Single nucleotide polymorphism
ST: Stable tansfer
VO_2_max: Maximum oxygen uptake
VO_2_peak: Peak oxygen uptake
